# Reply to “Finding the middle way: rethinking cGMP for sterility testing of cellular therapy products in minimal manipulation settings”

**DOI:** 10.1128/jcm.01372-25

**Published:** 2025-11-13

**Authors:** Anna F. Lau

**Affiliations:** 1 Sterility Testing Service, Clinical Center, National Institutes of Health 24481, Bethesda, Maryland, USA; Vanderbilt University Medical Center, Nashville, Tennessee, USA

**Keywords:** sterility testing, cGTP, cGMP, minimally manipulated products, cell therapy

## REPLY

We thank Fenwick et al. (1) for their thoughtful comments describing the need for nuance in the application of sterility testing for minimally manipulated cellular therapy products manufactured in hospital-based cell therapy laboratories (CTLs) in response to Gebo et al. (2). We agree that the overarching principles of current Good Manufacturing Practice (cGMP) are relevant to clinical microbiology laboratories performing sterility testing on more than minimally manipulated products classified under Section 351 (see examples in reference 3, Table 1) regulated through 21 CFR Part 610. This is emphasized by the U.S. Food and Drug Administration (FDA), which states that “the testing laboratory environment should employ facilities and controls comparable to those used for aseptic filling operation” (4), and by industry leaders that provide guidance for phase-appropriate adoption of cGMP and quality systems (5) (Fig. 1 in reference 2). Seperate to cGMP, we concur that discretion and better clarity may be needed regarding microbiological lab requirements for the sterility testing of minimally manipulated products regulated solely under 21 CFR Part 1271 and under Public Health Statutes, Section 361 (see examples in reference 3, Table 1), subject to current Good Tissue Practice (cGTP) regulated through 21 CFR 1271.

Specifically, for Section 361 minimally manipulated products, the underlying question is whether the quality systems employed in clinical microbiology laboratories certified by the Clinical Laboratory Improvement Amendments of 1988 (CLIA), regulated under 42 CFR Part 493 with compliance oversight by the Center for Medicare and Medicaid Services (CMS), are sufficient to satisfy cGTP requirements regulated under 21 CFR Part 1271 by the U.S. FDA. This question is particularly relevant for clinical microbiology laboratories that may only incubate sterility cultures (i.e., product inoculation into culture media is performed in the CTL with no open product manipulation in the microbiology laboratory). As mentioned in the concluding paragraph of reference 2, this question is being actively evaluated by a working group that includes members of the American Society for Microbiology (ASM) clinical microbiology community and members of the Association for the Advancement of Blood and Biotherapies (AABB, an international organization that sets standards, accredits facilities, and provides education in transfusion medicine and biotherapies). The aim of this working group is to evaluate CLIA and cGTP regulations and expectations to identify areas of overlap, address gaps in quality systems and practices, and clarify what CLIA-certified clinical microbiology laboratories need in order to fulfill the cGTP requirements for sterility testing of minimally manipulated Section 361 products.

We appreciate that the differences in regulatory oversight by two different agencies (CMS for CLIA laboratories and FDA for cGTP/cGMP laboratories), coupled with lessons learned and shared by the National Institutes of Health (NIH) following fungal contamination events in 2015, have led to increased awareness and hesitancy among the clinical microbiology community to take on sterility testing. For the contamination events at the NIH, the BACTEC blood culture system failed to flag positive culture bottles that had grown mold that was visible to the naked eye from two different products over a seven month period. Subsequent studies have noted that clinical blood culture systems, which have a limited temperature range of 35°C–37°C because they are primarily designed for the detection of bloodstream infections, are not sensitive for detecting mold contamination, as fungi prefer lower-temperature incubation and have slower CO_2_ respiration that may not trigger the positive alarm on clinical blood culture systems (3, 6, 7). Fenwick et al. states that separate fungal cultures may be excessive and counterproductive (1); however, the AABB standards for cellular therapies define sterility testing as “bacterial aerobic and anaerobic cultures with fungal culture” (8). Additionally, United States Pharmacopeia <71> (USP <71>) and USP <72> specify low-temperature incubation between 20°C and 25°C to improve fungal detection sensitivity (9, 10). Inclusion of “robust” fungal culture using appropriate media and incubation conditions is ultimately a risk-based decision that takes into consideration product exposure, process manipulation, and environmental monitoring to ensure that the product is safe to release for patient infusion. This may vary by facility and product. Notably, prior to 2015, sterility testing at the NIH for both Section 361 and Section 351 products had been performed in the CLIA-certified clinical microbiology laboratory for >25 years. Importantly, there was no specific documentation and no knowledge transfer during faculty turnover stating that non-CLIA-regulated testing was being performed in-house. Subsequently, there was no awareness amongst faculty regarding the historical commitment of the microbiology laboratory to overarching FDA regulations and quality principles governing cGTP and cGMP testing. Since the contamination events in 2015, the NIH has hosted numerous FDA inspections of manufacturing centers and the cGMP microbiology laboratory. The cGMP microbiology laboratory has also responded to multiple “requests for information” from the FDA regarding sterility testing practices and quality systems. Validation packets for method suitability testing and validation to demonstrate equivalency of the chosen NIH sterility method to USP <71> for each specific product have also been reviewed and discussed with the FDA. For these reasons, we share lessons learned to help other academic medical institutions and clinical microbiology laboratories navigate the differences in regulatory landscapes and expectations if choosing to perform product sterility testing in-house.

In turn, we appreciate that the messaging of sending out testing to cGMP labs has caused frustration among hospital-based CTLs due to reduced testing availability, delayed turnaround time, increased costs, and more complicated logistics. The goal of the ASM/AABB working group is to address these challenges, specifically for Section 361 minimally manipulated products, and to outline compliance needs and responsibilities for both manufacturers and testing labs. Importantly, some centers manufacture both Section 361 and Section 351 products. Section 351 products fall under cGMP, which is more rigorous than cGTP. In those instances, it may be difficult for a microbiology laboratory to differentiate quality practices for cGTP and cGMP; therefore, the more stringent cGMP requirements and practices may take precedence.

Additionally, while USP <71> (9) is the gold standard method for sterility testing and its test methods are harmonized across the United States, European, and Japanese pharmacopeias, a new chapter, USP <72> (10), became official on 1 August 2025. USP <72> is the pharmacopeia test standard for the use of respiration methods (e.g. blood culture systems) for sterility testing of short-life products. Use of blood culture systems for sterility testing of minimally manipulated 361 products, such as hematopoietic stem cells, is common practice in clinical microbiology laboratories (1, 3). USP <72>, written in collaboration with government liaisons representing the FDA and the NIH, can be used to address sterility testing analytical requirements for short-life products and is key for providing clarity regarding validation requirements such as method suitability testing at <10 CFU organism, media selection, incubation temperature, and length of incubation, all of which are different from the requirements written in USP <71>. Because this USP chapter is numbered <1,000, it is considered compendial (i.e., gold standard not requiring demonstration of alternative method equivalency) once it has been referenced in overarching USP documents (https://www.uspnf.com/notices/gc-72-73-faq). The timing for the cross-reference of USP <72> in overarching USP documents is not known currently (H. Tu, USP, personal communication).Therefore, as of now, from an FDA regulatory perspective, use of any non-USP<71> method (such as blood culture systems) for sterility testing of sterile drug products is considered an alternative method that must be validated according to USP <1223> (11) to demonstrate equivalency to USP <71>. Use of blood culture systems for sterility testing of short-life products as per USP <72> can be utilized once USP <72> has been cross-referenced in overarching USP documents.

In summary, we are aligned with the concerns raised by Fenwick et al. and are committed to providing clarity on requirements specific to Section 361 products for hospital-based CTLs, taking into consideration the perspectives of all contributing parties. Generating dialog, partnership, and mutual understanding of the risks and benefits between the manufacturing laboratory and the testing laboratory is key. Understanding the similarities and differences among CLIA, cGTP, and cGMP requirements will foster transparency and shared knowledge of expected practices and regulatory compliance for all stakeholders contributing to the delivery of safe, lifesaving products.

## References

[1] Fenwick A, Misra A, Martin I. 2025. Finding the middle way: rethinking cGMP for sterility testing of cellular therapy products in minimal manipulation settings. J Clin Microbiol. doi:10.1128/jcm.01358-2541230971

[2] Gebo JET, Feehely KM, Lattimore CE, Mogavero DP, Lau AF. 2025. Mini-review: equipment, software, and system validation (IOPQ) requirements for sterility testing of cellular therapies. J Clin Microbiol 63:e01477-24. doi:10.1128/jcm.01477-24 40788093 PMC12421869

[3] Cundell T, Atkins JW, Lau AF. 2023. Sterility testing for hematopoietic stem cells. J Clin Microbiol 61:e0165422. doi:10.1128/jcm.01654-22 36847535 PMC10035301

[4] United States Food and Drug Administration. 2004. Guidance for industry sterile drug products products by aseptic processing - current good manufacturing practice. White Oak, MD United States Food and Drug Administration

[5] Parenteral Drug Association. 2016. PDA Technical Report No. 56 Revised 2016 (TR 56) application of phase-appropriate quality system and cGMP to the development of therapeutic protein drug substance (API or biological active substance). Bethesda, MD Parenteral Drug Association

[6] England MR, Stock F, Gebo JET, Frank KM, Lau AF. 2019. Comprehensive evaluation of compendial USP<71>, BacT/Alert Dual-T, and Bactec FX for detection of product sterility testing contaminants. J Clin Microbiol 57:e01548-18. doi:10.1128/JCM.01548-18 30541938 PMC6355548

[7] Putnam NE, Lau AF. 2021. Comprehensive study identifies a sensitive, low-risk, closed-system model for detection of fungal contaminants in cell and gene therapy products. J Clin Microbiol 59:e0135721. doi:10.1128/JCM.01357-21 34406794 PMC8525565

[8] Association for the Advancement of Blood & Biotherapies. 2021. Standards for cellular therapy services. 10th ed. Association for the Advancement of Blood & Biotherapies, Bethesda, MD.

[9] United States Pharmacopeia. 2022. USP <71> Sterility tests. Rockville, MD United States Pharmacopeia

[10] United States Pharmacopeia. 2025. USP <72> Respiration-based microbiological methods for the detection of contamination in short-life products. Rockville, MD United States Pharmacopeia

[11] United States Pharmacopeia. 2022. USP <1223> Validation of Alternative MIcrobiological Methods. Rockville, MD United States Pharmacopeia

